# Intratumor Regulatory Noncytotoxic NK Cells in Patients with Hepatocellular Carcinoma

**DOI:** 10.3390/cells10030614

**Published:** 2021-03-10

**Authors:** Alessandra Zecca, Valeria Barili, Danila Rizzo, Andrea Olivani, Elisabetta Biasini, Diletta Laccabue, Raffaele Dalla Valle, Carlo Ferrari, Elisabetta Cariani, Gabriele Missale

**Affiliations:** 1 Unit of Infectious Diseases and Hepatology, Laboratory of Viral Immunopathology, Azienda Ospedaliero-Universitaria of Parma, 43126 Parma, Italy; alessandrazecca.az@gmail.com (A.Z.); oliver82a@gmail.com (A.O.); ebiasini@ao.pr.it (E.B.); carlo.ferrari@unipr.it (C.F.); 2 Department of Medicine and Surgery, University of Parma, 43126 Parma, Italy; barili.valeria@gmail.com (V.B.); danila.rizzo@studenti.unipr.it (D.R.); dilettal@hotmail.com (D.L.); raffaele.dallavalle@unipr.it (R.D.V.); 3 Independent Researcher, 41126 Modena, Italy; ecariani@hotmail.com

**Keywords:** natural killer cells, hepatocellular carcinoma, tumor microenvironment

## Abstract

Previous studies support the role of natural killer (NK) cells in controlling hepatocellular carcinoma (HCC) progression. However, ambiguity remains about the multiplicity and the role of different NK cell subsets, as a pro-oncogenic function has been suggested. We performed phenotypic and functional characterization of NK cells infiltrating HCC, with the corresponding nontumorous tissue and liver from patients undergoing liver resection for colorectal liver metastasis used as controls. We identified a reduced number of NK cells in tumors with higher frequency of CD56^BRIGHT^CD16^−^ NK cells associated with higher expression of NKG2A, NKp44, and NKp30 and downregulation of NKG2D. Liver-resident (CXCR6^+^) NK cells were reduced in the tumors where T-bet^hi^Eomes^lo^ expression was predominant. HCCs showed higher expression of CD49a with particular enrichment in CD49a^+^Eomes^+^ NK cells, a subset typically represented in the decidua and playing a proangiogenic function. Functional analysis showed reduced TNF-α production along with impaired cytotoxic capacity that was inversely related to CXCR6^−^, T-bet^hi^Eomes^lo^, and CD49a^+^Eomes^+^ NK cells. In conclusion, we identified a subset of NK cells infiltrating HCC, including non-liver-resident cells that coexpressed CD49a and Eomes and showed reduced cytotoxic potential. This NK cell subset likely plays a regulatory role in proangiogenic function.

## 1. Introduction

Natural killer (NK) lymphocytes are innate immune cells that are known for their capacity to kill transformed or virus-infected cells [1,2,3].

Besides their cytotoxic function, NK cells release cytokines such as TNF-α and IFN-γ that can control virus infection. In addition NK cells are involved in the maturation of dendritic cells [4,5] and display regulatory functions on the immune response by killing antigen-specific T cells [6] and stellate cells to limit liver fibrosis. The secretion of angiogenic factors to promote placental growth has been reported by specific organ-resident NK cells known as decidual NK cells (dNK) [7]. 

The liver is considered a preferential tissue for NK cell residency. In humans, almost 50% of all intrahepatic lymphocytes are NK cells. Intrahepatic NK cells are characterized by high cellular heterogeneity, and CD56^DIM^ and CD56^BRIGHT^ NK cells are present at similar frequencies in contrast to what is observed in the periphery. Liver-tissue-resident NK cells were first defined as CD56^BRIGHT^CXCR6+ cells, but more recently, the T-bet^lo^Eomes^hi^ phenotype has been identified as the peculiar liver-resident NK cell population, which is totally absent in peripheral blood [8,9,10].

The phenotype of human liver CD56^BRIGHT^ NK cells has been better described by the constitutive expression of the chemokine receptors CXCR6 and CCR5 and the tissue residency marker CD69. The CD56^BRIGHT^/CCR5^+^/CXCR6^+^/CD69^+^ phenotype seems to identify human liver-resident NK cells that also appear to be heterogeneous in their development [11]. Liver-resident NK cells play central roles in finalizing liver immune response in both physiological and pathological conditions [11].

NK cells not only contribute to the early innate immune response but also act as modulators of T cell responses directly enhancing or suppressing T cells functions [12]. Moreover, the liver is a solid organ whose parenchyma is constantly challenged by a plethora of different antigens drained from the gut every day. NK cells can also play a role in the defense against the development of liver fibrosis, limiting stellate cell activation and transdifferentiation to myofibroblasts, the major extracellular matrix-producing cell in the fibrotic liver. This mechanism is related to the upregulation on stellate cells of different ligands of the activating receptor NKG2D that trigger NK-cell-dependent killing [13,14]. 

A peculiar subset of organ-resident NK cells (dNK) has been identified in the human decidua. Human dNK acquire two crucial functions in early healthy pregnancy that differentiate them from peripheral or classical tissue-resident NK cells. dNK promote vascular growth through the production of proangiogenic factors, such as vascular endothelial growth factor (VEGF), placental growth factor (PlGF), angiopoietin 1, angiopoietin 2, and TGF-β1. In addition, dNK are able to release some chemokines (IL-8 and IP-10) that induce the migration of the extravillous cytotrophoblast, resulting in the invasion of spiral arteries thus contributing to the uterine vascular remodeling crucial for the placental development and for the outcome of pregnancy [15,16,17]. 

The role of NK cells in fighting cancer has been demonstrated both in experimental models and in humans. Immunotherapeutic strategies aimed at enhancing NK cell response are ongoing [18,19]. However, evidences indicate that immune cells not only contribute to tumor eradication but also initiate and promote cancer development. The tumor immune microenvironment, by several mechanisms, can polarize immune cells, switching from providing an effector and antitumor function to supporting tumor growth, including angiogenesis [20,21,22,23]. Despite the fact that this pattern is well established in other immune cells [24], little is still known about NK cell polarization in cancer, particularly in hepatocellular carcinoma (HCC). 

HCC is the most common type of liver cancer and is known for its poor prognosis [25]. The clinical outcome in patients treated by surgical resection or ablative treatment has been associated with the strength and quality of NK cell response [26,27,28,29]. Despite the knowledge about NK functions and phenotype in liver tissue [30,31,32], the role of NK cells in HCC remains unclear. This prompted us to investigate the characteristics of NK cells in these patients and in particular at the tumor site. A better understanding of tumor-infiltrating NK cell phenotypes may create the conditions to manipulate with appropriate strategies the immune response in these patients in order to control disease recurrence or progression, with a positive impact on survival.

In the present study, we employed an approach based on phenotypic characterization and functional assessment of HCC-infiltrating NK cells. This strategy allowed us to identify a subset of non-liver-resident, tumor-infiltrating NK cells that show a regulatory phenotype and could support tumor progression and neoangiogenesis.

## 2. Materials and Methods

### 2.1. Patients and Biological Samples

Liver and tumor sample fragments from 11 patients with HCC arising in Hepatitis C virus (HCV) infection, 6 patients with alcohol-related HCC, one patient with NASH-related HCC, and 7 patients undergoing liver resection for colorectal metastasis as control were obtained at surgery. Phenotypic and functional analysis of intrahepatic NK cells was performed on 12 paired HCC–nontumorous samples and controls, and 8 paired HCC–nontumorous samples were used to evaluate the production of proangiogenic factors (NK cells from 2 patients were both tested for phenotypic characterization and production of proangiogenic factors). Briefly, liver and tumor samples were fragmented and digested with resection buffer (PBS1x + 2% FCS+ 2 mM EDTA) supplemented with collagenase (50 mg/mL) (Sigma) and DNase (1 mg/mL) (Sigma-Aldrich, St. Louis, MO, USA) at 37 °C in a water bath for 30 min under stirring. After digestion, the samples were filtered with a Cell Strainer (70 µm, Falcon). The filtrated was selected separately by centrifugation on Ficoll-Hypaque density gradient centrifugation. The ring of lymphomononuclear infiltrating cells at the interface between Ficoll and supernatant was collected and washed with Hank’s Balanced Salt Solution (HBSS). Cells were cryopreserved in liquid nitrogen until analysis. 

HCC diagnosis was made by ultrasonography and computed tomography or magnetic resonance imaging in selected cases. Hepatitis B surface antigen (HBsAg) and antihuman immunodeficiency virus were negative in all cases. All patients were in Child–Pugh class A. HCC patients were in early stage (Barcelona Clinic Liver Cancer Stage A). The study population additional information are shown in Table 1.

The study was approved by the local ethical committee (Comitato Etico dell’Area Vasta Emilia Nord (AVEN), Italy). All participants gave written informed consent to participate in the study.

### 2.2. Phenotypic Analysis of NK Cells

After thawing, tumor- and liver-infiltrating lymphomononuclear cells were suspended in RPMI-1640 containing 8% human serum and stained with monoclonal antibodies specific for CD3-Alexa Fluor 700 (BioLegend, San Diego, CA, USA) and CD56-PE-Vio615 (Miltenyi Biotec, Bergisch Gladbach, Germany) in order to distinguish NK cells in the various samples (normal liver infiltrating NK cells, NLINK; tumor infiltrating NK cells, TINK; NK cells infiltrating the surrounding nontumorous liver, LINK). Infiltrating NK cells were characterized by a combination of monoclonal antibodies identifying activating and inhibitory receptors, such as NKG2A (CD94)-PerCP-Cy5.5 (BD Bioscience, Franklin Lakes, NJ, USA), NKG2D-PE (Thermo Fisher Scientific, Waltham, MA, USA), NKp30-Alexa Fluor 488 (R&D System, Minneapolis, MN, USA), NKp44-PEVio770 (Miltenyi Biotec, Bergisch Gladbach, DE), and CD16-FITC (BD Bioscience, Franklin Lakes, NJ, USA).

NLINK, TINK, and LINK were characterized by specific surface markers for maturation and liver residency, including CD27-FITC (BD), CD11b-APC-Cy7, CD49a-PE-Cy7, and CXCR6-PE-Dazzle594 (BioLegend, San Diego, CA, USA) in addition to CD56-PerCp (BD Bioscience, Franklin Lakes, NJ, USA) and CD3-Alexa Fluor 700 (BioLegend, San Diego, CA, USA). For the detection of intracellular antigens, cells were fixed and permeabilized with Fixation Permeabilization Concentrate and Diluent and Permeabilization Buffer (Thermo Fisher Scientific, Waltham, MA, USA) following the manufacturer’s instructions and stained with Eomes-PE and T-bet-APC (Invitrogen, Carlsbad, CA, USA).

To determine the content of cytotoxic molecules, infiltrating NK cells were incubated at room temperature (RT) with live/dead vitality dye (Invitrogen, Carlsbad, CA, USA). Subsequently, the cells were stained with surface antibodies CD3-PE-Cy7, CD16-FITC (BD Bioscience, Franklin Lakes, NJ, USA) and CD56-Pe-Vio615 (Miltenyi Biotec, Bergisch Gladbach, Germany) and then fixed with Cytofix solution (BD Bioscience, Franklin Lakes, NJ, USA). After the fixation step, the cells were permeabilized with Cytoperm solution (BD Bioscience, Franklin Lakes, NJ, USA) and labeled with the antibodies perforin-PerCp710 (Invitrogen, Carlsbad, CA, USA), granzyme B-AlexaFluor647 (BD Bioscience, Franklin Lakes, NJ, USA), and CD3ζ-PE (Invitrogen, Carlsbad, CA, USA).

All determinations were performed on an 8-fluorescence flow cytometer (FACS Canto II, Becton Dickinson (BD) Immunocytometry System, CA, USA), and the data were processed with the FACS Diva software (BD) and FlowJo software. The frequency of CD56^DIM^ and CD56^BRIGHT^ NK cells was defined for each patient, evaluating different fluorescence intensity of CD3^−^CD56^+^ cells, and expression of each marker was considered in total, CD56^DIM^, or CD56^BRIGHT^ cell populations, both as percentage and as median fluorescence intensity (MFI). For each marker, at least 5000 NK cells were gated.

### 2.3. Functional Analysis of NK Cells

IFN-γ and TNF-α production by NK cells was evaluated in all samples available for phenotypic analysis after overnight stimulation with or without IL-12 and IL-18 (5 ng/mL) in the presence of 10 µg/mL of brefeldin A (BFA) added during the last 3 h of culture. Surface staining with anti-CD3-PE, anti-CD56-PE-CF594, and anti-CD16-FITC (BD Bioscience, Franklin Lakes, NJ, USA) was performed. Then, cells were fixed with medium A reagent and permeabilized with medium B reagent (Nordic Mubio, Lifespan Biosciences, Seattle, WA, USA) in accordance with manufacturer’s instructions. Cytokine determinations were performed by intracellular cytokine staining (ICS) with anti-IFN-γ-PerCp-Cy5.5 (BioLegend, San Diego, CA, USA) and anti-TNF-α-APC (BioLegend, San Diego, CA, USA) monoclonal antibodies and analyzed by flow cytometry. The CD107a degranulation assay was performed to assess the cytotoxic potential. Cells were stimulated overnight with IL-12 and IL-18 (as above), then incubated with K562 target cells for the last 4 h in the presence of BFA and anti-CD107a-PE-Cy7 (BD Bioscience, Franklin Lakes, NJ, USA).

Data are expressed as the difference between the percentage of cytokine or CD107a-positive^+^ NK cells in the stimulated and unstimulated samples.

### 2.4. Proangiogenic Factor Analysis of CD49a + Eomes + NK Cells

The production of proangiogenic factors by NK cells was studied in 8 HCC patients (paired LINK and TINK) after overnight stimulation with IL-12 and IL-18 (5 ng/mL) in the presence of 10 µg/mL of brefeldin A (BFA). Surface staining with anti-CD3-BUV805, anti-CD56-BUV805, and anti-CD49a-BV510 (BD Bioscience, Franklin Lakes, NJ, USA) was performed. Cells were then fixed and permeabilized with Fixation Permeabilization Concentrate and Diluent and Permeabilization Buffer (Thermo Fisher Scientific, Waltham, MA, USA) following the manufacturer’s instructions and stained with Eomes-PE-Cy7 (Invitrogen), Angiopoietin 1 (ANGPT1)-Alexa Fluor750 (Bioss Antibodies, Boston, MA, USA), CXCL10/IP-10-Alexa Fluor 700 (R&D System, Minneapolis, MN, USA), Osteopontin-eFluor660 (Thermo Fisher Scientific, Waltham, MA, USA), IL-8-PerCp-eFluor710 (Thermo Fisher Scientific, Waltham, MA, USA), MMP-9-Alexa Fluor 488 (Abcam, Cambridge, UK), Placental growth Factor (PlGF)-Alexa Fluor594 (Bioss Antibodies, Boston, MA, USA), VEGF-PE (R&D system, Minneapolis, MN, USA), and Angiogenin-Alexa Fluor405 (Novus Biotechne, R&D System, Minneapolis, MN, USA). Analysis was performed on an 18-fluorescence flow cytometer (FACS Fortessa, Becton Dickinson (BD) Immunocytometry System, CA, USA). Data are expressed as the percentage of cytokine-positive cells in the CD49a+Eomes+ NK cell subset.

### 2.5. t-Distributed Stochastic Neighbor Embedding (tSNE) Analysis

The dimension reduction algorithm tSNE was applied to concatenated flow cytometry data from all TINK and LINK samples using default parameters (iterations, 1000; perplexity, 20; and θ, 0.5) in FlowJo. tSNE was applied to expression data for CD3, CD56, NKG2D, NKG2A, NKp44, NKp30, granzyme B, perforin, and CD3ζ.

### 2.6. Statistical Analysis

Statistical analysis was carried out using GraphPad Prism (version 7, San Diego, CA, USA) software. After analysis of variance (F test) and of normality (Kolmogorov–Smirnov test), a Mann–Whitney U test, Wilcoxon matched pairs test or paired t-test, and Spearman’s rank order correlation were applied as appropriate. Significant differences are marked in all figures. All tests were performed two-tailed, and *p* < 0.05 was considered significant.

## 3. Results

### 3.1. Activating and Inhibitory Receptors of Infiltrating NK Cells

The characteristics of the intrahepatic NK cells compartment were studied in paired liver and HCC samples obtained at surgical resection and in normal liver tissue surrounding colorectal metastases as a control.

**Figure 1:**
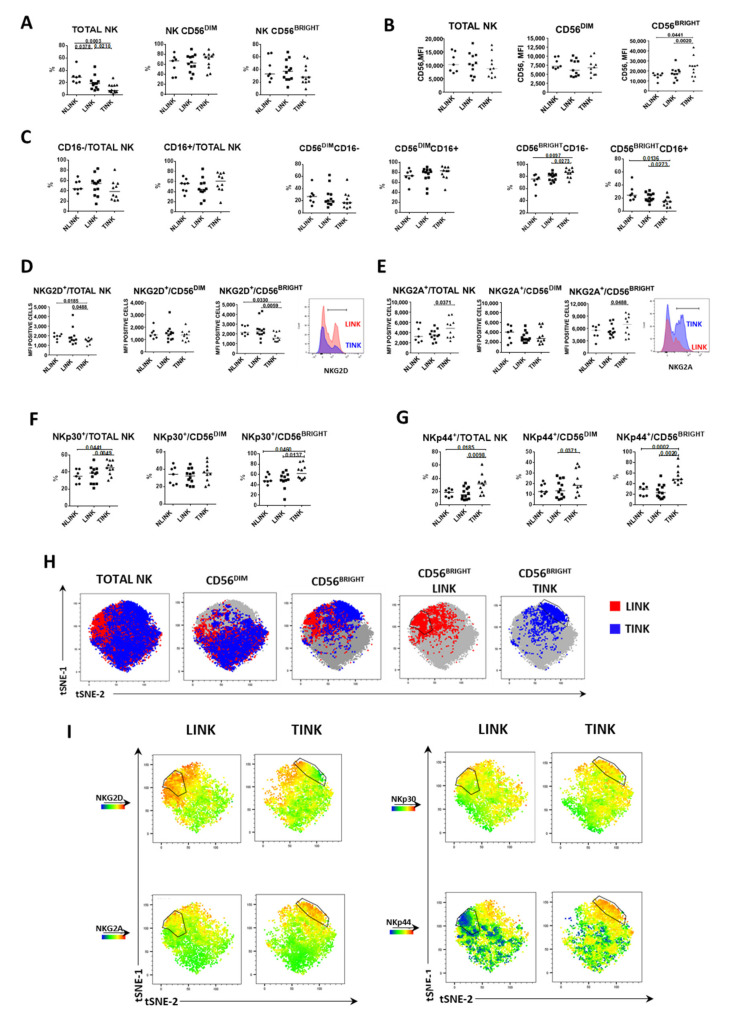
Frequency and phenotype of natural killer (NK) cells infiltrating normal liver tissue (NLINK), HCC-surrounding liver (LINK), and HCC (TINK). (**A**). Frequency of total NK cells and CD56^DIM^ and CD56^BRIGHT^ subpopulations in NLINK (n. 7), LINK (n. 12), and TINK (n. 12). (**B**). Median fluorescence intensity (MFI) of CD56 expression in NK cell subsets (total NK, CD56^DIM^, and CD56^BRIGHT^) from NLINK, LINK, and TINK. (**C**). Percentage of cells expressing CD16 in NK cell subsets. D, E. MFI of NKG2D (**D**) and NKG2A (**E**) expression in NK cell subsets (Total NK, CD56^DIM^ and CD56^BRIGHT^) from NLINK, LINK, and TINK. F, G. Expression of NKp30 (**F**) and NKp44 (**G**) in NK cell subsets from NLINK, LINK, and TINK. Horizontal lines represent median values. Statistics by Wilcoxon matched pairs test (LINK vs. TINK) and Mann–Whitney test (NLINK vs. LINK and NLINK vs. TINK). (**H**). The dimensionality reduction algorithm t-distributed stochastic neighbor embedding (tSNE) was applied to flow cytometry data on NK cells (single-cell expression values from total live lymphocytes for CD3, CD56, NKG2A, NKG2D, NKp30, and NKp44) to generate a two-dimensional map of NK cells from paired TINK and LINK from all the experimental samples. tSNE analysis shows the segregation of NK subsets in study groups. The black line shows the CD56^BRIGHT^ subset showing different expression patterns in LINK and TINK. (**I**). tSNE colored by expression intensity of NK cell receptors NKG2A, NKG2D, NKp30, and NKp44 in LINK and TINK samples. Upregulation is represented in red and downregulation in blue, with intermediate color shades representing intermediate levels.

Intrahepatic NK cells were identified by the strategy shown in Supplementary Figure S1. Briefly, after exclusion of cell aggregates, gating on lymphocytes, and subsequent exclusion of dead cells, NK cells were identified as CD3^−^CD56^+^. The percentage of NK cells on total lymphocytes infiltrating tumor samples (TINK, n. 12) was significantly decreased compared to surrounding liver samples (LINK, n. 12) and to normal liver samples (NLINK, n. 7). NK cells were also more represented in the normal liver compared to LINK. CD56^BRIGHT^ and CD56^DIM^ NK subsets were similarly represented among different tissues (Figure 1A). TINK exhibited a higher median fluorescence intensity (MFI) of CD56 molecule compared to LINK and NLINK, especially in the CD56^BRIGHT^ subset (Figure 1B).

Infiltrating NK cells were analyzed for the expression of inhibitory and activating receptors. A higher frequency of CD56^BRIGHT^CD16^−^ NK cells was detected in tumor samples with respect to LINK and NLINK (Figure 1C). Activating NKG2D receptor was expressed at a lower level in TINK compared to LINK and NLINK, both in total NK and in CD56^BRIGHT^ subpopulation. Conversely, inhibitory NKG2A (CD94) expression was higher in NK cells infiltrating HCC compared to the nontumorous counterpart, both in total NK and in CD56^BRIGHT^ subset (Figure 1D,E). Further analysis highlighted a higher number of TINK positive for both NKp30 and NKp44 receptors compared to LINK and NLINK, both in total NK and in CD56^BRIGHT^ NK cells (Figure 1F,G).

To visualize the relative intensity of NK cell receptor expression, we performed dimension reduction by tSNE (t-distributed stochastic neighbor embedding) on multiparametric flow cytometry data of NKG2D, NKG2A, NKp30, and NKp44 from the LINK and TINK groups (Figure 1H,I). Differential distribution of events was evident in particular for the CD56^BRIGHT^ subset among LINK (red) and TINK (blue) (Figure 1H). In Figure 1I, the color shades represent the expression level of different phenotypic markers, red indicating upregulation and blue downregulation. In particular, the TINK CD56^BRIGHT^ subpopulation showed lower expression of NKG2D, but higher expression of NKG2A, NKp30, and NKp44 compared to LINK.

### 3.2. Phenotypic Analysis of Liver Residency, Development, and Maturation Markers

**Figure 2:**
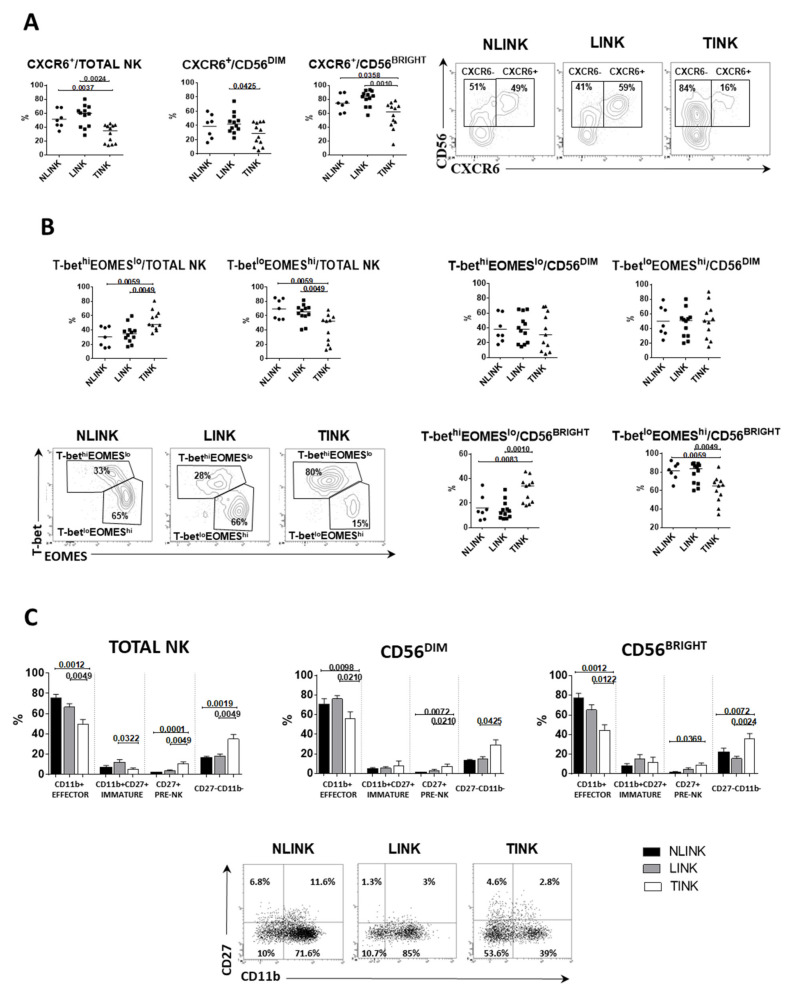
Frequency and residency phenotypes of NK cells infiltrating normal liver tissue (NLINK, n. 7), HCC-surrounding liver (LINK, n. 12), and HCC (TINK, n. 12). (**A**). Left: percentage of CXCR6+ NK cells in NK cell subsets from NLINK, LINK, and TINK. Right: representative dot plots of CXCR6 staining. (**B**). Frequency of NK cells expressing different levels of T-bet and Eomes in total, CD56^DIM^, and CD56^BRIGHT^ NK cells. Dot plots represent T-bet and Eomes staining. (**C**). Percentages of NK cells expressing different levels of CD11b and CD27. Lower panels: dot plots of CD11b and CD27 staining. Horizontal lines represent median values; bar graphs show mean ± SEM. Statistics by Wilcoxon matched pairs test (LINK vs. TINK) and Mann–Whitney test (NLINK vs. LINK and NLINK vs. TINK).

**Figure 3:**
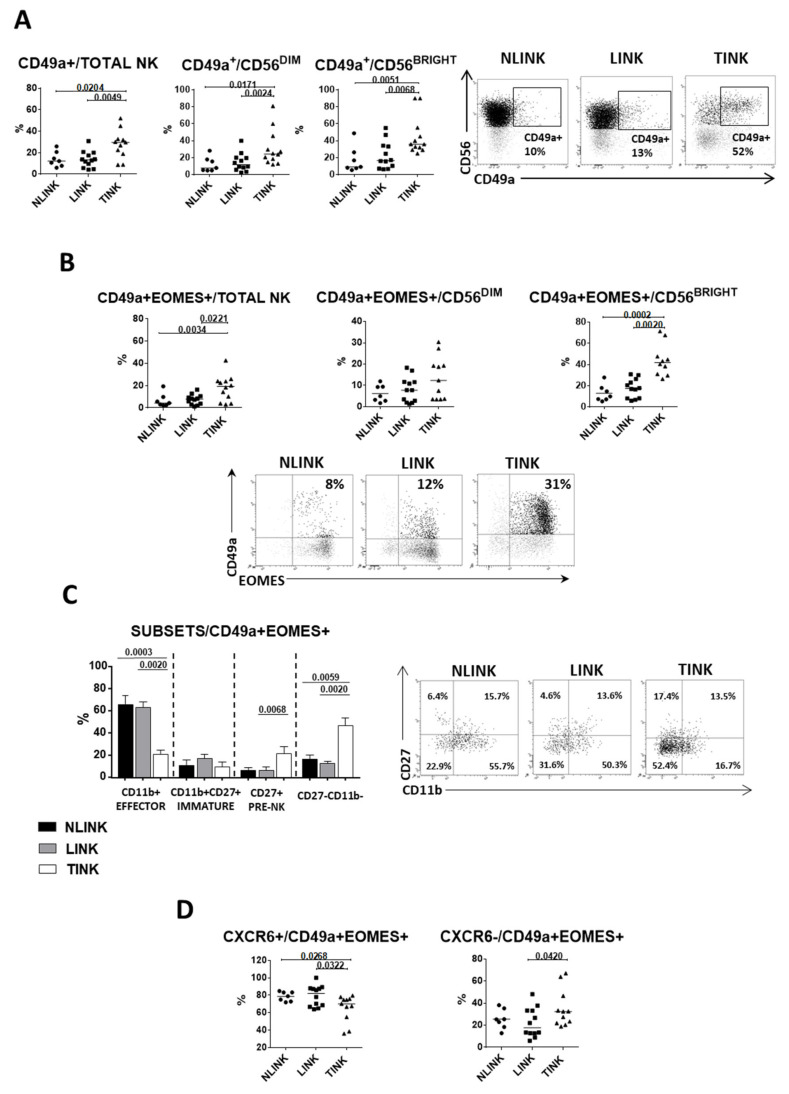
Regulatory NK cells in hepatocellular carcinoma. (**A**). Percentage of CD49a^+^ cells in NK cell subsets (total NK, CD56^DIM^, and CD56^BRIGHT^) from NLINK (n. 7), LINK (n. 12), and TINK (n. 12). Representative dot plots of CD49a^+^NK cells in NLINK, LINK, and TINK are shown on the right side. (**B**). Upper panels: percentage of CD49a^+^Eomes^+^ in NK cell subsets (total NK, CD56^DIM^, and CD56^BRIGHT^) from NLINK, LINK and TINK. ***Lower panels:*** representative dot plots of CD49a and Eomes staining. (**C**). Left panel: percentage of NK cells expressing CD11b and CD27 in regulatory (CD49a^+^ and Eomes^+^) NK cells. Right panel: representative dot plots. (**D**). Percentage of CD49^+^Eomes^+^ NK cells expressing CXCR6 in NLINK, LINK, and TINK. Horizontal lines represent median values; bar graphs show mean ± SEM. Statistics by Wilcoxon matched pairs test (LINK vs. TINK) and Mann–Whitney test (NLINK vs. LINK and NLINK vs. TINK).

NK cells expressing chemokine receptor CXCR6 (liver-resident NK cells) [33] were significantly less frequent in TINK compared to both LINK and NLINK. The multiplicity of CXCR6^+^ TINK was lower among all NK cell subsets (total NK, CD56^BRIGHT^, and CD56^DIM^) (Figure 2A), suggesting that a portion of TINK may be recruited from the periphery or locally expanded from non-liver-resident NK cells. CD49a represents another tissue residency marker for NK cells, which has been shown to be associated with poor HCC prognosis [34]. A significantly higher frequency of CD49a^+^ cells was also observed in TINK (Figure 3A).

To better understand development and maturation of TINK, we evaluated the combined expression of T-bet/Eomes and CD27/CD11b. Peripheral blood NK cells are known to show a prevalent T-bet^hi^Eomes^lo^ profile, while T-bet^lo^Eomes^hi^ is the prevalent phenotype in the intrahepatic compartment [33]. More than 50% of NLINK and LINK were T-bet^lo^Eomes^hi^, while TINK showed a higher frequency of T-bet^hi^Eomes^lo^ NK cells that were particularly evident in the CD56^BRIGHT^ subset (Figure 2B).

Based on CD27 and CD11b expression, human NK cells can be divided in four subgroups related to NK cells development: CD27+ (CD27 single positive (SP), pre-NK), CD27 + CD11b + (DP, immature), CD11b + (CD11b SP, effector) and CD27-CD11b- (double negative (DN), regulatory) [35].

TINK cells showed a higher frequency of CD27^+^CD11^−^ (pre-NK) cells and CD27^−^CD11b^−^ (regulatory) cells compared to NLINK and TINK in both CD56^DIM^ and CD56^BRIGHT^ subpopulations (Figure 2C).

The coexpression of CD49a and Eomes (particularly within the CD56^BRIGHT^ subset) is typical of decidual regulatory NK cells that play a role in angiogenesis and placenta development [35]. CD49a^+^Eomes^+^ NK cells were significantly more represented in TINK than in LINK and NLINK. (Figure 3B). Within this CD49a^+^Eomes^+^ subset, CD27^+^CD11b^−^ and CD27^−^CD11b^−^ phenotypes were prevalent in TINK with respect to LINK and NLINK (Figure 3C), similarly to what was reported for decidua NK cells [36,37]. Interestingly, the prevalence of the CD49a^+^Eomes^+^CXCR6^−^ phenotype was higher in TINK than in LINK (Figure 3D), suggesting that a quota of non-liver-resident TINK may play a regulatory or proangiogenic function in the tumor microenvironment.

### 3.3. Functional Analysis of Infiltrating NK Cells

**Figure 4:**
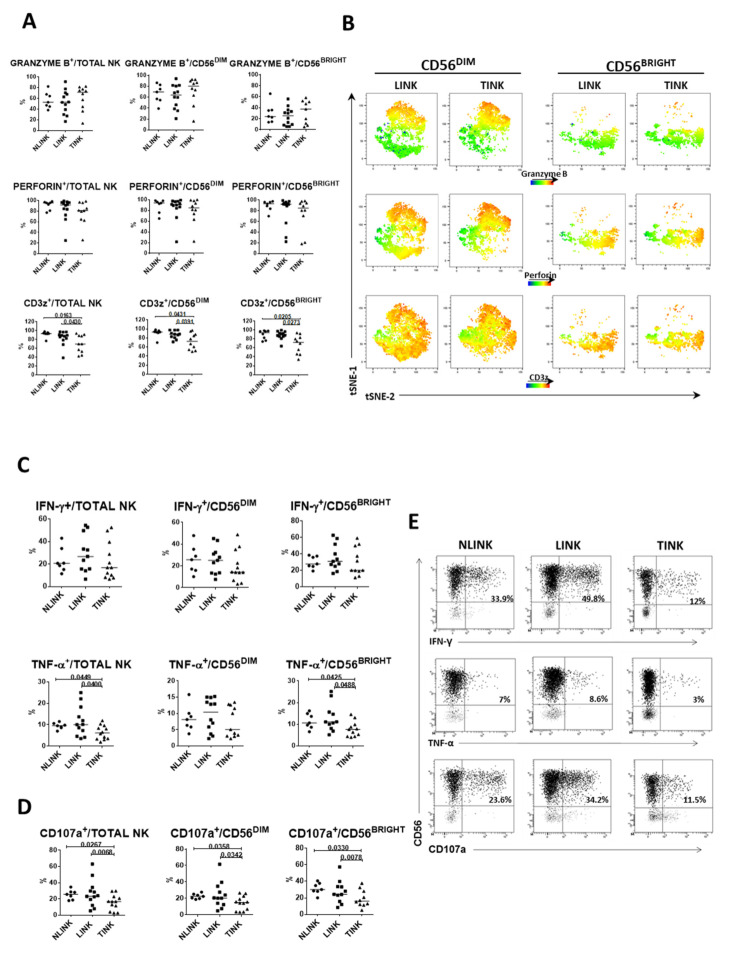
Infiltrating NK cell functional analyses. (**A**). Percentage of granzyme B+ (upper panels), perforin+ (middle panels), and CD3ζ+ (lower panels) cells in NK cell subsets from NLINK (n. 7), LINK (n. 12), and TINK (n. 12). (**B**). The dimensionality reduction algorithm tSNE was applied to flow cytometry data (single-cell expression values from total live lymphocytes for CD3, CD56, perforin, granzyme B, and CD3z) to generate a two-dimensional map of NK cells from paired TINK and LINK. tSNE analysis shows the segregation of NK cell subsets in LINK and TINK. Color intensity represents expression levels (red: upregulation; blue: downregulation). Cytokine production (**C**) and cytotoxic potential (CD107a expression (**D**) in NK cell subsets from NLINK, LINK, and TINK after stimulation with IL-12 and IL-18. Data are expressed as the difference between the percentage of cytokine+ or CD107a+ NK cells in the stimulated and unstimulated samples. (**E**). Representative dot plots showing functional capacity of infiltrating NK cells. Horizontal lines represent median values; statistics by Wilcoxon matched pairs test (LINK vs. TINK) and Mann–Whitney test (NLINK vs. LINK and NLINK vs. TINK).

The expression of molecules associated with cytotoxic response (granzyme B and perforin) was similar in TINK, LINK, and NLINK. In contrast, CD3ζ expression was significantly reduced in TINK compared to LINK and NLINK, particularly in CD56^BRIGHT^ NK cells (Figure 4A). We performed a dimension reduction by tSNE on multiparametric flow cytometry data to observe the relative intensity of granzyme B, perforin, and CD3ζ expression in TINK and LINK (Figure 4B). Differential distribution of events in the tSNE map could be observed as a different level of expression only of the CD3ζ marker.

Functional assessment showed a similar level of IFN-γ production in TINK, LINK, and NLINK, while TNF-α production (Figure 4C, lower panels) and degranulation (CD107a expression) (Figure 4D) were significantly reduced in TINK compared to LINK and NLINK in total NK cells and CD56^BRIGHT^ and, limited to CD107a, also in CD56^DIM^ NK cells (Figure 4D).

In order to evaluate the function of different TINK subpopulations identified by phenotypic analysis, we analyzed the degranulation efficiency (CD107a^+^) and cytokine (IFN-γ and TNF-α) production, focusing on non-liver-resident NK cells (T-bet^hi^Eomes^lo^ and CD49a^+^Eomes^+^ NK cells).

**Figure 5:**
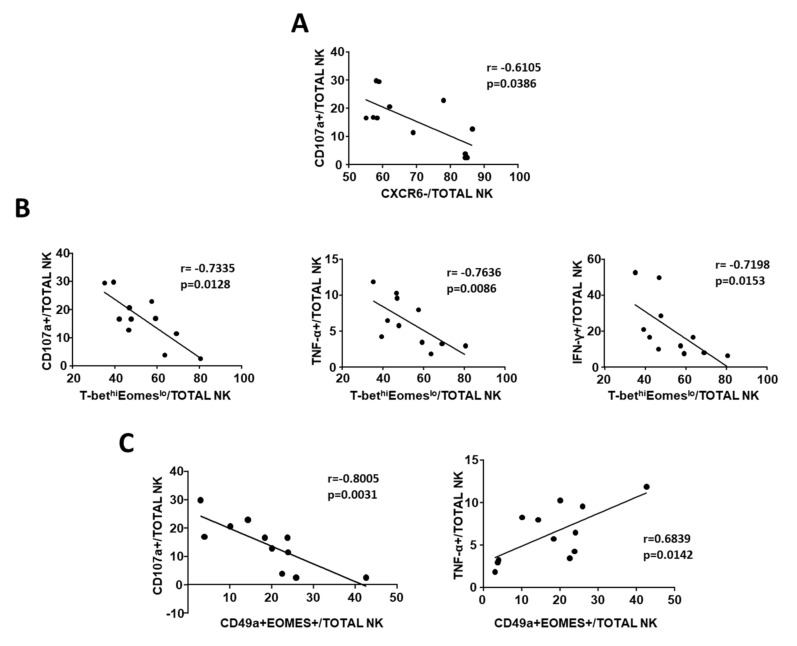
Correlation between phenotypes and TINK function. (**A**). Correlation between frequency of CXCR6^−^ NK cells and CD107a expression in TINK (n. 12). (**B**). Correlation between percentage of CD107a^+^, IFN-γ^+^, and TNF-α^+^ TINK and frequency of T-bet^hi^Eomes^lo^ NK cells. (**C**). Correlation between frequency of CD49a^+^Eomes^+^ (regulatory) TINK and degranulation capacity (left) or TNF-α production (right). Statistics by Spearman’s rank order correlation.

The percentage of CXCR6^−^ cells was inversely correlated with CD107a expression (Figure 5A), suggesting a degranulation defect in CXCR6^−^ cells that represent more than 50% of TINK. In contrast, the ability to produce IFN-γ and TNF-α was unrelated to the prevalence of CXCR6^−^ cells (data not shown). Interestingly, the percentage of T-bet^hi^Eomes^lo^ NK cells was inversely correlated with the expression of CD107a, IFN-γ, and TNF-α (Figure 5B).

The multiplicity of CD49a^+^Eomes^+^ NK cells, which are believed to express regulatory functions, was inversely correlated with degranulation capacity, while a positive correlation was found with TNF-α production, suggesting that these cells do not exert a cytotoxic function even though they can actively produce cytokines (Figure 5C).

### 3.4. CD49a^+^Eomes^+^ NK Cell Subset Shows Proangiogenic Features in HCC

**Figure 6:**
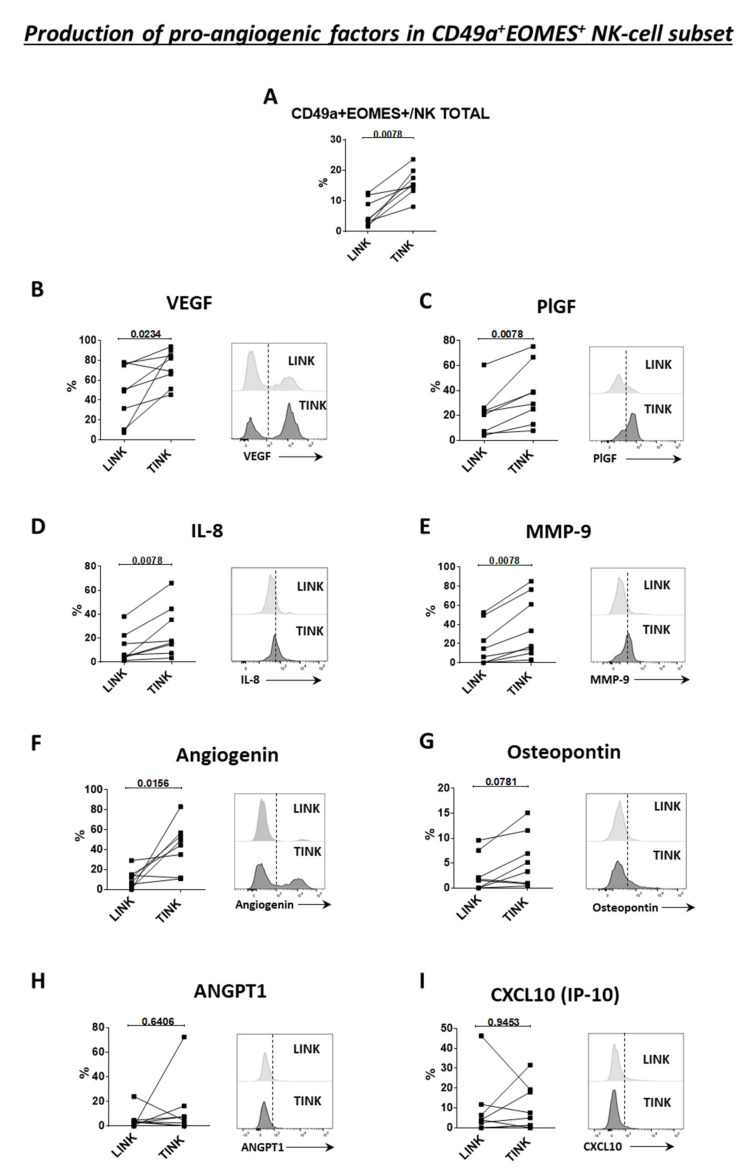
Production of proangiogenic factors by CD49a^+^Eomes^+^ NK cells in LINK and TINK (n. 8). (**A**). Frequency of CD49a^+^Eomes^+^ in total NK cells in the tumor and liver. VEGF (**B**), PlGF (**C**), IL-8 (**D**), MMP-9 (**E**), angiogenin (**F**), osteopontin (**G**), ANGPT1 (**H**), and CXCL10 (**I**) producing cells upon IL-12 and IL-18 stimulation. On the right side of each panel are representative histograms showing the MFI of each cytokine. Statistics by Wilcoxon matched pairs test.

Eight patients with HCC arising in HCV-related (four subjects) and alcohol-related (four subjects) liver disease were tested. First, we confirmed a higher frequency of CD49a^+^Eomes^+^ NK cells in the tumor compared to the surrounding nontumorous liver tissue (Figure 6A). Then, we analyzed the production of angiogenic factors by this subset. Tumor-derived CD49a^+^Eomes^+^ NK cells could produce significantly higher levels of pro-angiogenic factors compared to liver-derived CD49a^+^Eomes^+^ NK cells. In particular, significantly higher frequencies of VEGF, placental growth factor (PlGF), IL-8, MMP-9, and angiogenin-producing NK cells were present in the tumor (Figure 6B–F), while the frequency of angiopoietin 1 (ANGPT1), osteopontin, and CXCL10 (IP-10)-positive NK cells was similar between the two compartments (Figure 6G–I). Comparisons were carried out by paired analysis (Wilcoxon matched pairs test).

In order to better understand the relevance of these findings, we compared the production of angiogenic factors by CD49a^+^Eomes^+^ NK cells to that of NK cells not coexpressing CD49a and Eomes. Only VEGF was more expressed by CD49a^+^Eomes^+^ cells in nontumorous liver tissue, while in the tumor, CD49a^+^Eomes^+^ cells expressed higher levels of VEGF, PlGF, IL-8, MMP-9, angiogenin, and CXCL10 (IP-10) (Supplementary Figure S2A,B). Finally, the frequency of NK cells not coexpressing CD49a and Eomes and producing different angiogenic factors was similar in the two compartments (Supplementary Figure S2C).

## 4. Discussion

NK cells are the main effector cells of the innate immune system that are known to kill virally infected, transformed and stressed cells [2,3]. Data from the literature support the relevance of NK cells in controlling neoplastic progression, particularly in HCCs [26,38]. However, there is also ambiguity regarding the correlation between the multiplicity and the role of tumor-infiltrating NK cells and patient prognosis [27,39,40,41].

In this study, phenotypic characterization and functional assessment were carried out, comparing NK cells infiltrating HCC (TINK), surrounding nontumorous liver NK cells (LINK), and normal liver NK cells (NLINK).

Phenotypic analysis of TINK showed a lower frequency of total NK cells with enrichment of the CD56^BRIGHT^CD16^−^ subset characterized by higher expression (MFI) of CD56 molecules compared to control groups. This superbright phenotype has been described in tumor-associated NK cells as well as in decidual NK cells (dNK). CD56^SUPERBRIGHT^ cells may play a regulatory and proangiogenic function but can also represent NK cells with enhanced cytotoxic potential after activation by IL-15 priming [39,42].

In agreement with previous studies [43,44,45], NKG2D was downregulated with the concomitant NKG2A upregulation in TINK. Local production of soluble mediators, among which IL-10 and TGF-β promoted by hypoxia, may be responsible for modulation of these receptors [44,46,47]. This can contribute to the inability to perform relevant effector functions by intratumor NK cells, and it is associated with metastatic dissemination and poor prognosis in liver cancer [48,49,50].

Natural cytotoxicity receptors NKp30 and NKp44 were upregulated in TINK, in particular in the CD56^BRIGHT^ subset. Upregulation of the NKp30 receptor is in agreement with a recent study [51] that showed intratumor higher levels of NKp30 due to enrichment of its inhibitory isoform, responsible for a defective NKp30 function. In addition, in this case, expression may be influenced by cytokine milieu in the tumor microenvironment, which selects, through the epigenetic role of some cytokines (TGF-β, IL-15 and IL-18), the NKp30 inhibitory variant and affects NK cell cytotoxicity [43].

NKp44 is a peculiar receptor for many reasons. Expression of this molecule is typical of activated NK cells with high cytotoxic activity [52], but tumors can regulate NKp44 ligand expression to escape NK cell immune response, inducing expression by tumor ta-get cells of exosomal proliferating cell nuclear antigen (PCNA) when physically contacted by NK cells [53], thus explaining its high expression in CD56^DIM^ and ^BRIGHT^ subsets. In addition, NKp44 is constitutively expressed by decidual NK cells [7,54,55,56,57,58].

Tissue-infiltrating NK cells were also analyzed for differentiation and tissue residency markers. Peripheral blood NK cells are known to show a prevalent T-bet^hi^Eomes^lo^ profile, while T-bet^lo^Eomes^hi^ is the prevalent phenotype in the intrahepatic compartment, as confirmed by our NLINK and LINK analysis [9,33]. In contrast, TINK, and especially CD56^BRIGHT^ cells, showed a prevalence of the T-bet^hi^Eomes^lo^ phenotype similar to the circulating NK cell counterpart. This peculiar pattern may suggest that some of the tumor-infiltrating NK cells could have been recruited from the periphery or locally expanded from peripherally derived NK cells. Consistent with this interpretation, the liver residence marker CXCR6 was expressed in a significantly lower number of TINK compared to LINK and NLINK.

The characterization of CD27 and CD11b expression that has been used to describe NK cell maturation [35,59,60] showed significant enrichment in TINK of CD27^−^CD11b^−^ and CD27 single positive cells, subsets described as immature and regulatory NK cells with low cytotoxic activity [61,62].

We also showed in TINK an increase in cells expressing CD49a, which is a marker of regulatory functions and is associated with tumor progression. CD49a^+^ expression in NK cells has been shown to be positively related to the expression of inhibitory receptors, such as PD-1, LAG-3, and CD96, and negatively related to the expression of activating receptors, such as CD160, CD244, and NKG2D [34]. This suggests that CD49a expression may reflect a sort of *exhausted* phenotype for NK cells. Furthermore, it has been shown that patients with higher percentage of CD49a^+^ cells infiltrating the HCC were more likely to exhibit a more aggressive tumor phenotype characterized by neoplastic thrombus, absence of tumor capsule, and shorter disease-free and overall survival [34].

Coexpression of CD49a and Eomes may define a subset of NK cells with a regulatory function. In decidua, this NK cell subset produces a large amount of cytokines and chemokines, such as pleiotrophin, osteoglycin, and osteopontin, that regulate the immune condition and promote fetal development. [35,63,64]. In the present study, CD49a and Eomes coexpression was significantly higher in TINK, and this subset was enriched in CD27^−^CD11b^−^, CD27^+^CD11b^−^, and CXCR6^−^ NK cells. Moreover, we were able to demonstrate that this subset specifically produced more angiogenic factors specifically in the tumor. This suggests that these cells may favor tumor growth.

Overall, phenotypic analysis suggests a shift in TINK towards regulatory function, which was confirmed by functional analysis showing reduced CD3ζ expression and TNF-α production together with impaired degranulation and cytotoxic capacity, despite the conserved expression of cytotoxic molecules (perforin and granzyme B). Moreover, frequencies of CD49a^+^Eomes^+^, CXCR6^−^, and Tbet^hi^Eomes^lo^ NK cells were all correlated with a defect in cytotoxic function as suggested by low expression of CD107a. A higher frequency of Tbet^hi^Eomes^lo^ NK cells was also associated with reduced TNF-α and IFN-γ production, while CD49a^+^Eomes^+^-positive cells were associated with higher TNF-α production, suggesting a regulatory function of this subset.

Altogether, our results show that NK cells infiltrating the HCC show reduced expression of tissue residency markers and increased expression of differentiation markers represented in peripheral blood NK cells. Moreover, these NK cells are phenotypically and functionally similar to NK cells infiltrating decidua that are shifted toward non-cytotoxic regulatory functions [57,65] and may play a pro-oncogenic role. It has been shown that NK cells may promote tumor growth and vascularization of trophoblast choriocarcinomas [39] in non–small-cell lung cancer (NSCLC) and renal cell carcinoma, where NK cells can acquire the decidual-like CD56^bright^CD16- phenotype related to proangiogenic functions [66,67]. Our results are in favor of a similar role in HCC. The composition of HCC-infiltrating NK cells from our study is similar to what was reported for dNK cells; more than 50% are CD27-CD11b- and about 20% are CD27-SP, indicating an association with a high level of NKG2A and peculiar NKp44 upregulation, while NKG2D is downregulated and degranulation (CD107a) capacity is reduced [55,58,68,69]. 

In the present study, phenotypic and functional analysis strongly suggests that, beside a quantitative reduction of NK cells in the tumor tissue, a significant number of tumor-infiltrating NK cells do not contribute to fighting HCC but could play a different regulatory role supporting tumor growth. Specific subsets of NK cells in HCC could play a crucial role in tumor progression, sustaining endothelial proliferation and cell migration and promoting metastatic dissemination. This angiogenic profile of NK cells might provide a novel target for potential immunomodulating therapies, which are currently lacking for patients with HCC.

## Figures and Tables

**Table 1:** Study population.

	Healthy Controls	HCC Patients
#	7	18
Gender (M/F)	4/3	13/5
Mean age ± SEM (years)	68.1 ± 3.9	72.11 ± 1.74
BCLC (A-0/B)	na	13/5
Child-Pugh	na	A
HCC nodules, number (range)	na	1–3
Size of larger HCC (mean ± SEM, cm)	na	4.04 ± 0.36
alpha-feto protein	na	15.3 ± 4.96
Etiology	na	
- HCV		11
- Alcohol		6
- Nash		1

## Data Availability

The data presented in this study are available on request from the corresponding author. The data are not publicly available due to ethical reasons.

## Supplementary Materials

The following are available online at https://www.mdpi.com/2073-4409/10/3/614/s1, Figure S1: Dot plots representative of flow cytometry analysis. Figure S2: Production of proangiogenic factors between CD49a^+^Eomes^+^ and NK cells not expressing CD49a and Eomes in TINK and LINK.
