# Supplementary material for: Intratumor Regulatory Noncytotoxic NK Cells in Patients with Hepatocellular Carcinoma
Source: Cells. 2021 Mar 10;10(3):614. doi: 10.3390/cells10030614 (PMC7999652; doi:10.3390/cells10030614)
Supplement: Supplementary file 1 [file cells-10-00614-s001.pdf]

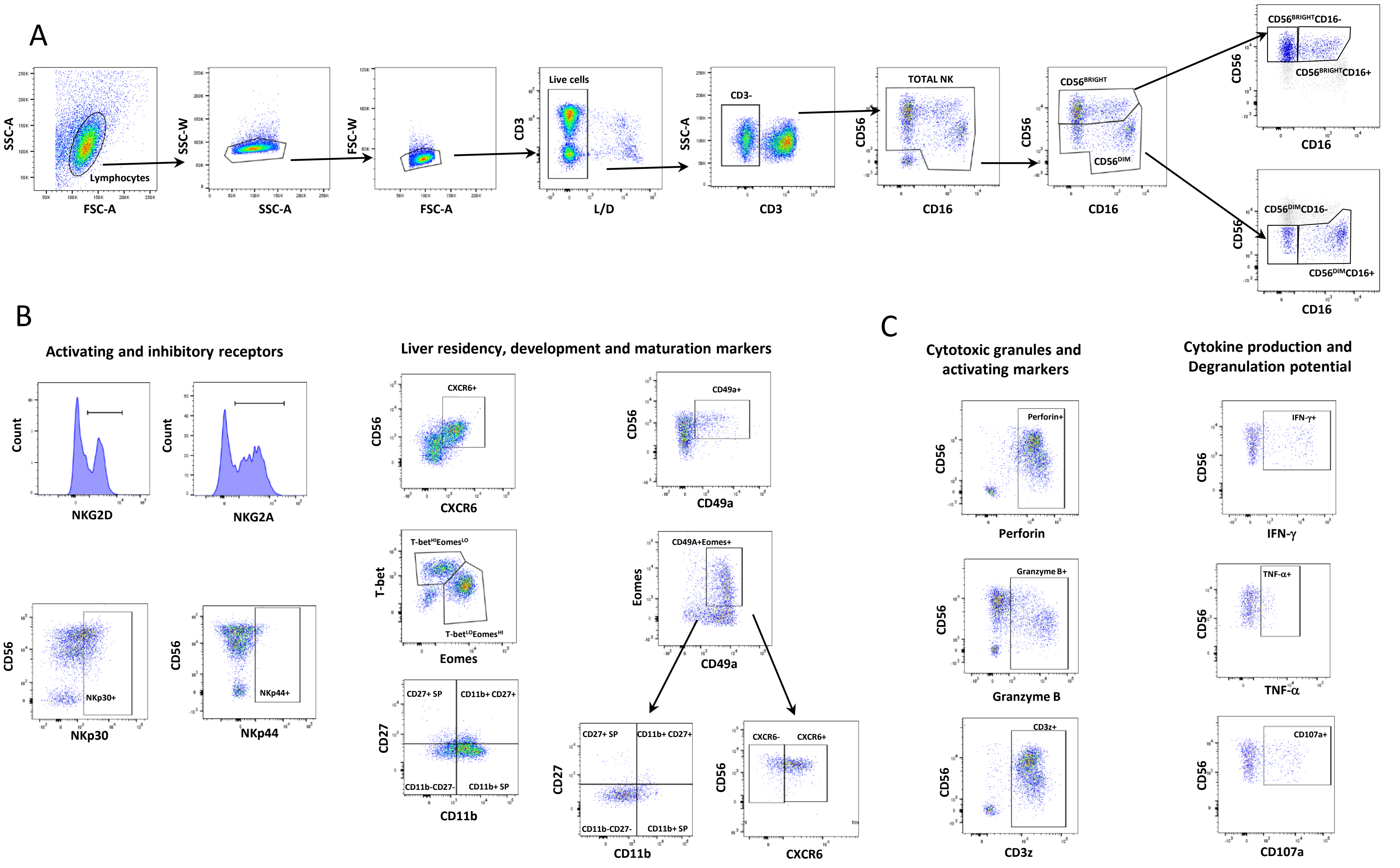

Supplementary Figure 1. ***Flow cytometry analysis representative dot plots*. A:** Gating strategy for the identification of infiltrating NK-cells (Lymphocytes, singlets, live cells, CD3-, total NK, $CD56^{BRIGHT}$ and $CD56^{DIM}$ and CD16+/-) using multicolor flow cytometry. **B:** Dot plots representing analysis example for each markers that have been used for phenotypical assessment (NKG2D, NKG2A, NKp30, NKp44, CXCR6, T-bet, Eomes, CD27, CD11b and CD49a) of NK-cells. **C:** Dot plots showing example of function analysis by Granzyme B, Perforin, CD3z, CD107a, TNF-α and IFN-γ staining.

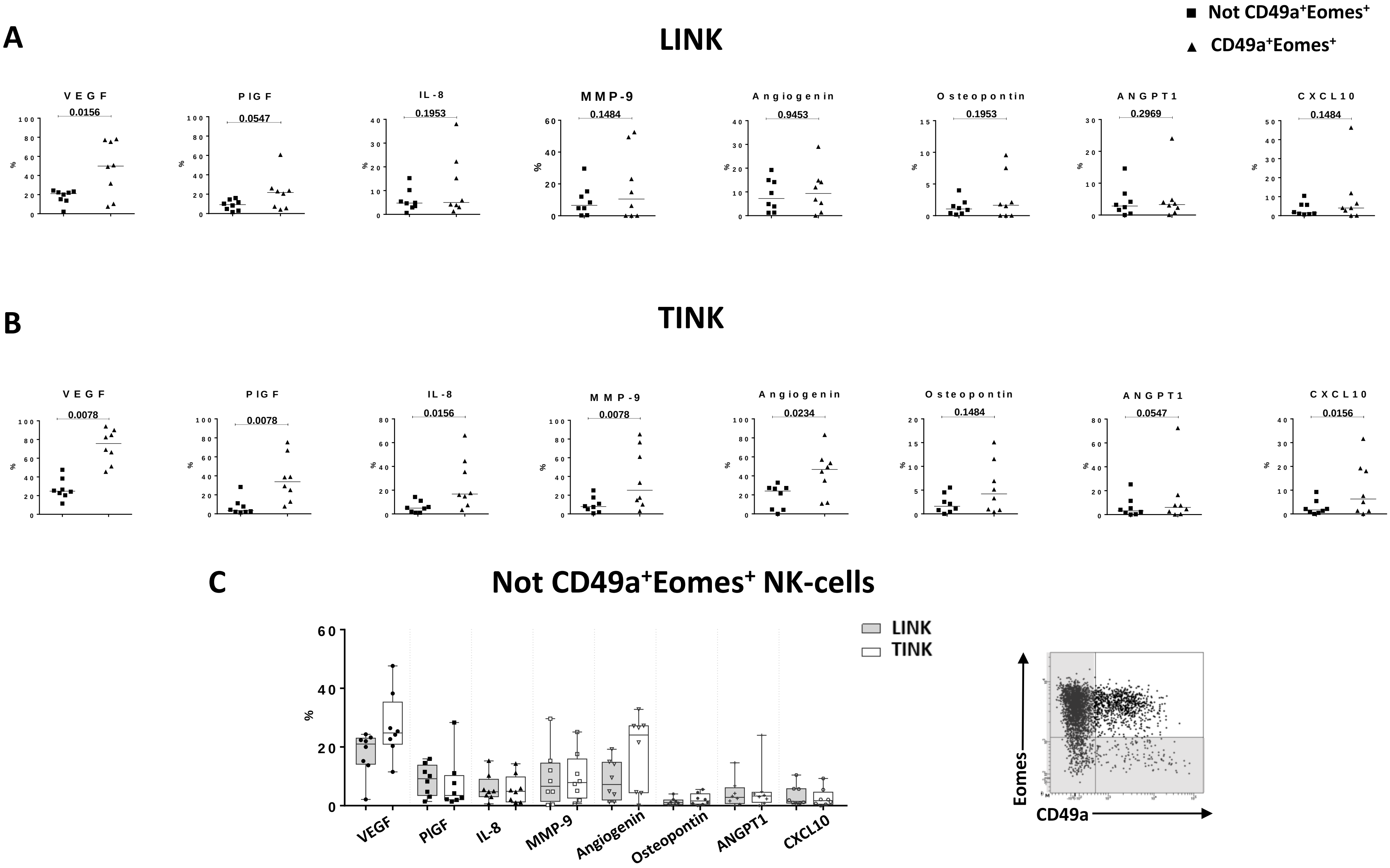

Supplementary Figure 2. ***Pro-angiogenic factors production by CD49a^{+}Eomes^{+} and NK-cells not co-expressing CD49a and Eomes.*** **A:** Frequency of cytokines positive cells in the two subsets (CD49a+Eomes+ and not CD49a+Eomes+) in the liver. **B:** Percentage of NK-cells producing angiogenic factors among CD49a+Eomes+ and NK-cells not co-expressing CD49a and Eomes in the tumor. **C:** Frequency of cytokines positive NK-cells not co-expressing CD49a and Eomes in the two compartments. On the right side, representative dot plot showing with grey background the not co-expressing CD49a and Eomes NK-cells. Pro-angiogenic factors staining was performed after overnight IL-12 and IL-18 stimulation. Horizontal lines represent median values, statistics by Wilcoxon matched pairs test (LINK vs TINK).
